# *Aedes aegypti* and *Ae. albopictus* microbiome/virome: new strategies for controlling arboviral transmission?

**DOI:** 10.1186/s13071-022-05401-9

**Published:** 2022-08-09

**Authors:** Marcela Gómez, David Martinez, Marina Muñoz, Juan David Ramírez

**Affiliations:** 1 Centro de Investigaciones en Microbiología y Biotecnología-UR (CIMBIUR), Facultad de Ciencias Naturales, Universidad del Rosario, Bogotá, Colombia; 2 Grupo de Investigación en Ciencias Básicas (NÚCLEO) Facultad de Ciencias e Ingeniería, Universidad de Boyacá, Tunja, Colombia; 3 Molecular Microbiology Laboratory, Department of Pathology, Molecular and Cell-Based Medicine, Icahn School of Medicine at Mount Sinai, New York, NY USA

**Keywords:** Arbovirus, ISV, Metagenomics, Microbiota, Vectors, Virome

## Abstract

**Abstract:**

*Aedes aegypti* and *Aedes albopictus* are the main vectors of highly pathogenic viruses for humans, such as dengue (DENV), chikungunya (CHIKV), and Zika (ZIKV), which cause febrile, hemorrhagic, and neurological diseases and remain a major threat to global public health. The high ecological plasticity, opportunistic feeding patterns, and versatility in the use of urban and natural breeding sites of these vectors have favored their dispersal and adaptation in tropical, subtropical, and even temperate zones. Due to the lack of available treatments and vaccines, mosquito population control is the most effective way to prevent arboviral diseases. Resident microorganisms play a crucial role in host fitness by preventing or enhancing its vectorial ability to transmit viral pathogens. High-throughput sequencing and metagenomic analyses have advanced our understanding of the composition and functionality of the microbiota of *Aedes* spp. Interestingly, shotgun metagenomics studies have established that mosquito vectors harbor a highly conserved virome composed of insect-specific viruses (ISV). Although ISVs are not infectious to vertebrates, they can alter different phases of the arboviral cycle, interfering with transmission to the human host. Therefore, this review focuses on the description of *Ae. aegypti* and *Ae. albopictus* as vectors susceptible to infection by viral pathogens, highlighting the role of the microbiota-virome in vectorial competence and its potential in control strategies for new emerging and re-emerging arboviruses.

**Graphical Abstract:**

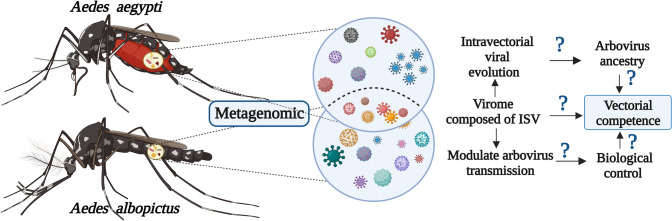

## Background

Vector-borne diseases significantly impact public health, affecting approximately 30% of the world's population [1–3]. In particular, viral pathogens transmitted to humans by insects—arboviruses—are one of the main concerns due to the accelerated increase in their incidence and geographical distribution in recent years [4–6], with dengue (DENV), Zika (ZIKV), and chikungunya (CHIKV) the arboviruses of most significant medical importance in the world and requiring active epidemiological surveillance [7–9]. DENV, the most prevalent viral infection in tropical and subtropical countries [10–14], infects between 100 and 400 million people per year, and its incidence has increased 30-fold in recent decades [15, 16]. Since 2000, CHIKV and ZIKV have spread and caused significant outbreaks in Asia and the Americas [17–19].

DENV, CHIKV, and ZIKV are transmitted to humans predominantly through the highly competent mosquitoes *Aedes aegypti* and *Aedes albopictus* [20, 21]. *Aedes aegypti* is a mosquito vector with a wide distribution worldwide, especially in tropical and subtropical environments, and is closely associated with urban areas and areas with environmental disturbances [22, 23]. Contrastingly, *Ae. albopictus* presents a greater geographical expansion [24]*,* colonizing all five continents [20]. Despite vector control efforts, in recent years an increase in the geographical distribution of *Aedes* spp. has been detected due to factors associated with climate change [5, 25–27], globalization [28–30], urbanization [29–31], and resistance to different insecticides [32]. As a result, these vector species are considered a serious threat [33], compromising the effectiveness of preventive measures, control programs, and the management of outbreaks of the diseases they are involved in.

Furthermore, the search and discovery of viruses in insect vectors have accelerated in the last decade, thanks to advances in metagenomic sequencing technologies [34–37]. However, studies on vector virome are still scarce [37], and the role of insect-specific viruses—ISVs (viruses that only replicate in arthropod cells)—is virtually unknown, despite the importance in the prevention and control of mosquito-borne diseases. Different investigations indicate that ISVs are closely related to families of pathogenic viruses such as *Flaviviridae* and *Togaviridae* [38, 39], where DENV, CHIKV, and ZIKV are found. Therefore, ISVs can become potential pathogenic viruses in invertebrates [40, 41]; on the contrary, they could be highly related to the competition of vectors to transmit arboviruses [42, 43], or serve in the future as biological control agents against known arboviruses [44, 45]. Knowledge about the composition of viral communities in mosquitoes of the genus *Aedes* will contribute to the understanding of the mosquito–virus–pathogen interaction, as well as to elucidating new control strategies in the face of new arboviral epidemics. This review focuses on the description of the composition and diversity of the microbiota-virome of vectors involved in the transmission of arboviruses such as *Ae. aegypti* and *Ae. albopictus*. Also, the role of these microbes in the modulation of vectorial capacity and in the potential strategy of biological control is highlighted.

### *Aedes aegypti* and *Aedes albopictus:* competent vectors in arboviral transmission

*Aedes aegypti* (*Stegomyia aegypti*) and *Ae. albopictus* (*Stegomyia albopicta*) are of interest primarily because of their association with emerging and re-emerging infectious diseases [17]. These two mosquito vectors have been described as highly competent in the transmission of arboviral pathogens such as DENV, ZIKV, and CHIKV [5, 22, 23, 29, 33, 46]. The species share several characteristics that give them adaptive advantages over others, making them successful invaders. The rapid spread and adaptation in tropical, subtropical, and temperate zones, and thus expansion of global coverage [5, 21, 26], may be related to large-scale epidemics and recent simultaneous outbreaks [20, 29]. *Aedes aegypti* is originally from Africa, considered a primary vector of some arboviruses. It has a high potential for pathogen transmission to humans due to its purely anthropophilic habits, reproduction in domestic (urban) and peridomestic environments, use of artificial containers as breeding places [5, 47], and greater availability of natural containers for oviposition [48, 49]. In comparison, *Ae. albopictus*, known as the tiger mosquito (Asian), is ecologically more flexible, with a more comprehensive geographical range than *Ae. aegypti* [24, 50]. It is found in suburban, rural, and sylvatic habitats, where it presents a wide range of hosts including humans, livestock, amphibians, reptiles, and birds [24, 29]. Both vectorial species present high ecological plasticity in heterogenous anthropic, climatic, and environmental conditions [25, 31, 50, 51]. Thus, *Ae. aegypti* and *Ae. albopictus* also reveal opportunistic feeding patterns in multiple human hosts during a gonotrophic cycle [52], diapause states (metabolism decreased at meager rates of energy expenditure and subsequent inactivity) during the development of eggs in drought conditions [53], resistance to insecticides such as DDT (dichlorodiphenyltrichloroethane) and pyrethroids [54], and versatility in the use of clean or stagnant water hatcheries in urban and natural environments [24].

*Aedes aegypti* and *Ae. albopictus* are considered two of the most invasive mosquito species [23, 50]. Competition between the two species in their ranges, whether native or invaded, frequently causes competitive displacement of one of the species [55], which can modify the epidemiology of arboviral diseases [56–58]. However, today, the two still coexist in large regions of the world [21, 59]. Several authors have revealed that the coexistence of *Ae. aegypti* and *Ae. albopictus* vector species in the same geographical areas can increase the risk of infection or co-infection for humans, especially during outbreaks or arboviral expansion [22, 26, 46, 60, 61]. Braks et al. [48] demonstrated that habitat is a determining factor in the abundance of the two species. Although *Ae. aegypti* predominates in urban areas and *Ae. albopictus* in rural areas, the two species can coexist in peri-urban areas, as demonstrated in several regions of Brazil and in the state of Florida in the United States. Thus, the segregation of different habitats may be a mechanism promoting coexistence between species, which avoids direct competition [55, 62].

Different factors influence the spatio-temporal relationships of virus–vector–human transmission, including vector capacity, vector competence, and host susceptibility [63]. Vector competence is defined as the intrinsic ability of the vector to successfully transmit a virus [63]. It normally comprises the capacity of a vector to acquire, maintain, and transmit a pathogen agent [64]. In contrast, vector capacity involves environmental factors such as temperature, vertebrate host availability, vector feeding behavior, population density, longevity, and predation [63]. Scientific evidence indicates that factors such as climate, vegetation, and building density affect the distribution of both species [5, 51, 57, 65], which determines the probability of arbovirus transmission in each region. In addition, virus–vector dynamics associated with the genetic and immunology background of each population of *Aedes* and the associated microbiota, including ISVs and arboviral variants, also play essential roles in the spread of the virus by vector species [23, 66, 67].

**Fig. 1:**
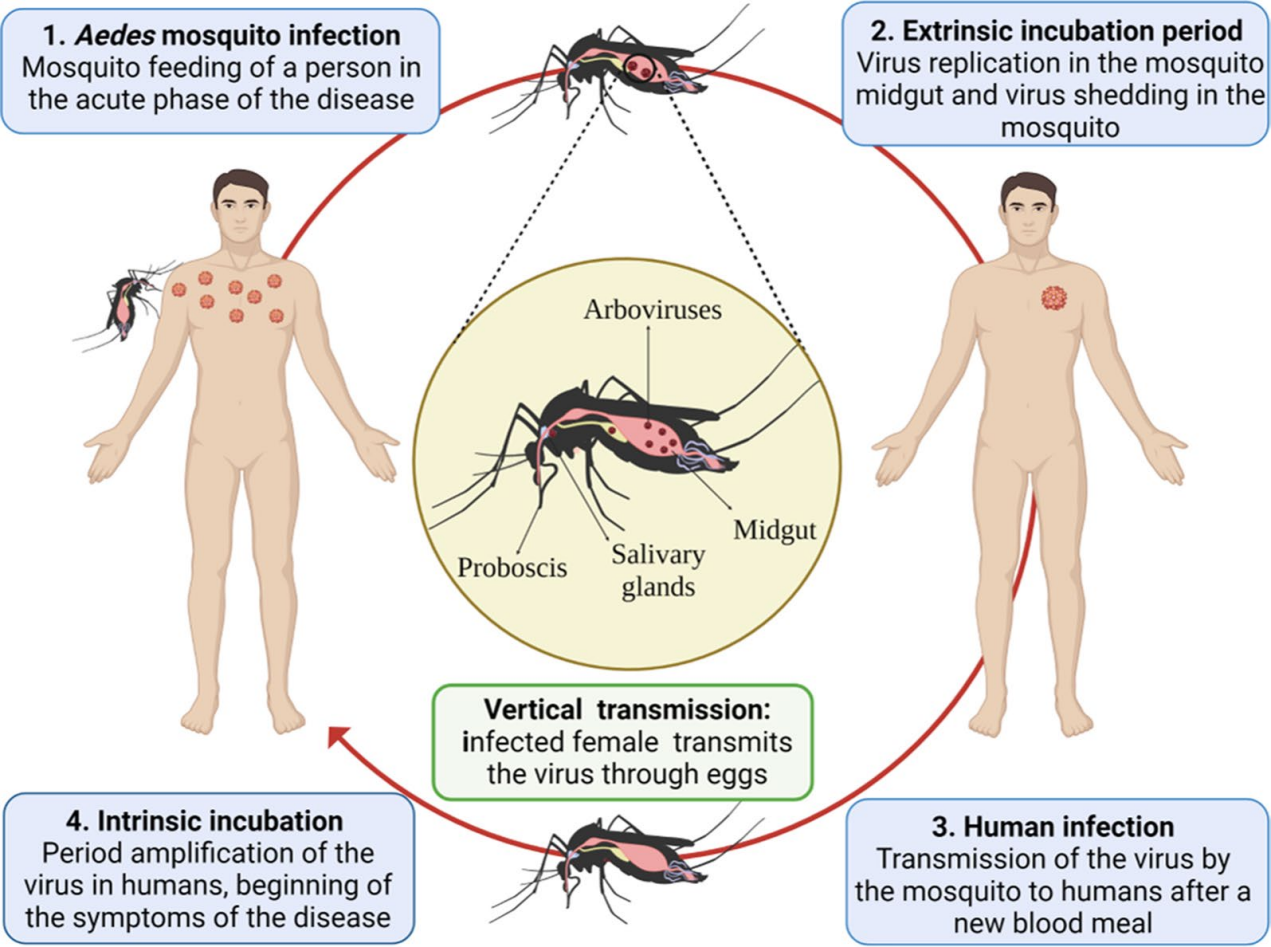
The urban cycle of arboviruses in humans and mosquitoes. Figure created with BioRender.com

In epidemic arboviruses such as DENV, ZIKV, and CHIKV, the urban (enzootic) cycle is essential for maintaining transmission without requiring other cycles to achieve disease persistence [8, 9, 15]. Transmission of arboviruses by vectors is carried out in various steps (Fig. 1): (1) infection of the vector (female mosquitoes) after feeding with blood from a host during the acute febrile or viremic phase of the disease [15]; (2) extrinsic incubation period, with the replication of the virus in the middle intestine of the mosquito and its spread to distal tissues, until it reaches the salivary glands (reservoir organs for the virus) [68, 69], a process influenced by the ambient temperature, the strain of the virus, immunological response of the mosquito, and the vector capacity [63]; (3) transmission of the virus from the infectious mosquito to a human during a new blood-feeding [9]; and (4) intrinsic incubation period and appearance of disease symptoms. Symptoms develop within an incubation period of 4 to 10 days after being bitten by an infected mosquito and usually last 2 to 7 days. The asymptomatic or symptomatic person can transmit the virus to a new mosquito and keep the epidemic cycle active [15, 33].

Interestingly, vertical or transovarial transmission, defined as the transfer of pathogens from the infected parent to part of the offspring [70], also occurs when the infected female mosquito transmits the virus through eggs [68] (Fig. 1). This phenomenon can occur especially during interepidemic periods and periods of drought [59, 71, 72]. In endemic areas, during unfavorable periods for horizontal transmission, the ability of some arboviruses to persist in the environment after long periods of few or no documented human cases is not clear [73, 74]. Although vertical transmission occurs at low rates, it can limit effective surveillance for infectious diseases and the achievement of comprehensive arboviral control. In particular, during the extrinsic incubation period, the genetic diversity of the virus population decreases stochastically as the virus crosses different anatomical barriers for transmission [69, 71], such as the midgut, where viral replication occurs in distal organs for dissemination, and salivary glands where transmission is finally ensured. Therefore, virus populations capable of overcoming these tissue barriers are considered to undergo positive or purifying selection where new genotypes may emerge [15, 40, 69], influencing vector competence in mosquitoes.

### Microbiota and vector competence in *Ae. aegypti and Ae. albopictus*

In recent years, studies on the biology of arbovirus-transmitting insects have shown that in addition to disease-causing pathogens, mosquitoes harbor many microorganisms such as bacteria, viruses, fungi, and parasites [23, 75–77]. It has been shown that microbial composition and functionality in mosquitoes are influenced by genetic factors of the host and the environment [78]. Therefore, it is believed that mosquito microbiomes can vary substantially among individuals, life stages, species, and geographical area [36, 79]. In *Ae. aegypti* and *Ae. albopictus*, there is evidence that acquisition of the microbiota is attributed primarily to trans-stage transmission from larva to adult and consumption of water, nectar, or other environmental food sources [35, 78, 80]. The vectorial competence, blood consumption, and infection by different pathogens may persistently or transiently change the bacterial composition through alterations in the redox state of metabolism. However, despite these differences, there appears to be a “core microbiota,” a collection of critical bacterial taxa that commonly colonize different mosquito species [35, 81], although the specific roles of these taxa and their relationship to vectorial competence remain unclear.

In the case of adult *Ae. aegypti* and *Ae. albopictus* mosquitoes, Proteobacteria, Bacteroides, Firmicutes, and Actinobacteria are the phyla that group more than 99% of the total components of the microbiota community [36]. Some of the bacteria associated with the different body organs of *Aedes* mosquito species are *Acetobacter*, *Burkholderia*, *Cupriavidus*, *Elizabethkingia*, *Escherichia-Shigella*, *Ochrobacterium*, *Pantoea*, *Serratia*, and *Sphingomonas* at the salivary gland level; *Asaia*, *Bacillus*, *Chryseobacterium*, *Chromobacterium*, *Cupriavidus*, *Enterobacter*, *Enterococcus*, *Klebsiella*, *Kluyvera*, *Leucobacter*, *Pantoea*, *Pichia*, *Pseudomonas*, *Serratia*, and *Sphingomonas* at the midgut level; and *Pseudomonas*, *Acinetobacter*, *Cupriavidus*, *Ochrobacterium*, *Stenotrophomonas*, and *Wolbachia* at the reproductive organ level [35, 36, 75, 82, 83]. Although the bacterial components of the mosquito microbiota are widely investigated [76, 84–86], some studies also include other entities such as fungi and yeasts identified from metagenomic shotgun sequencing [87] and targeted sequencing of the 28S marker (28S rRNA). In laboratory-reared and field-collected *Aedes*, mainly yeasts such as *Candida* and *Pichia* have been identified, as well as a variety of filamentous fungi such as *Penicillium* [35]. The protists of helminths comprising this microbiota and their influence on the insect physiology remain unknown.

**Fig. 2:**
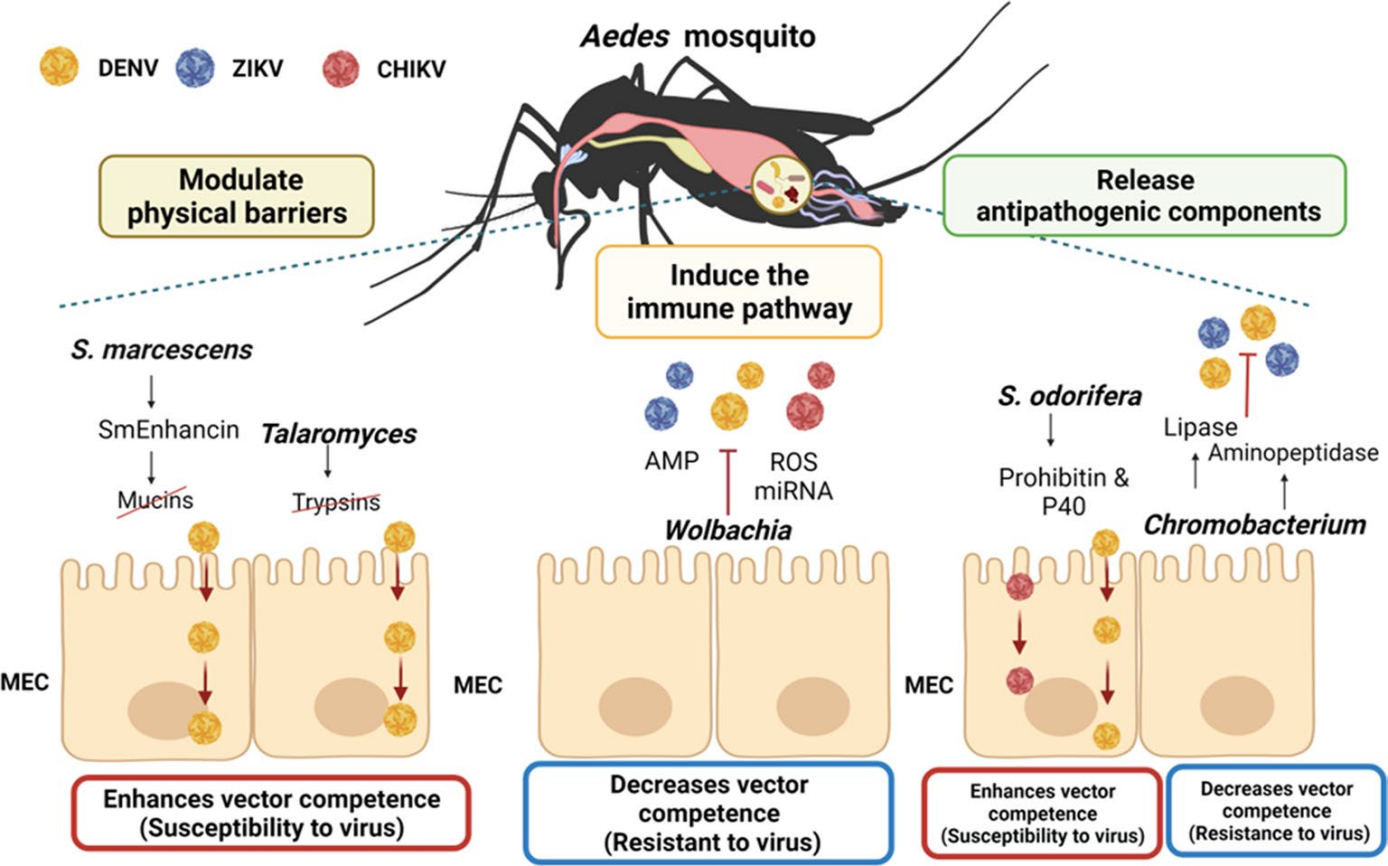
*Aedes* mosquito–microbiota–arbovirus interactions may modulate vectorial competence. Some viral regulatory strategies include the following: Modulation of physical barriers in midgut epithelial cells (MEC). *Serratia marcescens* by releasing Sm enhancin protein and the fungus *Talaromyces* by suppressing the expression of digestive enzymes (trypsin) in the midgut of *Aedes* can promote susceptibility to DENV infection. Activation of immune response signaling pathways and release of antipathogenic components. *Wolbachia* in the presence of arboviruses can induce antimicrobial peptides (AMP), melanization, and the production of reactive oxygen species (ROS), among others, that restrict arboviral activity. Release of antipathogenic compounds. *Chromobacterium* sp. Panama (Csp_P), by degrading the arbovirus coat protein, limits the replication of DENV and ZIKV, while *Serratia odorifera* participates in the interaction between P40 polypeptide and prohibitin, proteins associated with DENV and CHIKV infection in *Aedes*. Red boxes indicate interactions that increase vector competence (susceptibility to virus infection). Blue boxes indicate interactions that decrease vector competence (resistance to virus infection). *CHIKV* chikungunya virus, *ZIKV* Zika virus, *DENV* dengue virus, *ROS* reactive oxygen species, *AMP* antimicrobial peptides, *miRNA* microRNA, *MEC* midgut epithelial cells, *P40* polyvinentide P40, *Pr* prohibitin. Figure created with BioRender.com

Recent studies have shown that bacterial communities of adult *Ae. aegypti* and *Ae. albopictus* can play an essential role in regulating viral invasion by generating resistance or susceptibility to infection against arboviruses and other pathogens [64, 75, 84, 86, 88] (Fig. 2). Thus, some patterns of microbial regulation have been described that ultimately modulate the vectorial competence. Strategies of viral regulation include modulation of physical barriers in midgut epithelial cells (MEC), activation of immune response signaling pathways, and release of antipathogenic components. One of the symbionts that promote susceptibility to arbovirus infection is *Serratia marcescens*. This can degrade mucins bound to the intestinal membrane of *Ae. aegypti* by releasing the Sm enhancin protein, which decreases the natural protection of intestinal mucus [89] and thereby promotes the spread of pathogenic viruses such as DENV in the mosquito gut. Similarly, the fungus *Talaromyces* sp. in this same vector species can suppress the expression of digestive enzymes (trypsin) in the midgut of *Aedes* mosquitoes and, consequently, increase susceptibility to DENV infection [90] (Fig. 2).

*Wolbachia,* an intracellular symbiont prevalent in some insects, has been identified as capable of reducing vectorial competition by manipulating the reproduction of its host insects and generating cytoplasmic incompatibility [75]. Experimental trials show a significant reduction in the transmission of DENV, ZIKV, CHIKV, and yellow fever virus (YFV) in *Aedes* mosquitoes inoculated with this bacterium [67, 91]. Curiously, negative interactions between *Wolbachia* and arboviruses are mainly associated with enhanced insect immune response. This occurs by inducing antimicrobial peptides (AMP), melanization, and the production of reactive oxygen species (ROS) [84]. Likewise, it has been demonstrated that this microorganism, by inducing microRNA (miRNA) production, suppresses the expression of essential genes during viral genome methylation [92, 93]. In addition, *Wolbachia* can compete for resources by sequestering cholesterol and other lipids in insect cells [94], which ultimately limits arboviral infections (Fig. 2).

Interestingly, bacteria with broad-spectrum antipathogenic activity against arboviruses, such as *Chromobacterium* sp. Panama (*Csp_P*), can reduce DENV infection in *Ae. aegypti* by degrading the viral envelope protein through the production of a type of aminopeptidase [95, 96]. This bacterium can also restrict *Plasmodium falciparum* infection in *Ae. gambiae* through the antiparasitic protein romidepsin [97]. In contrast, *Serratia odorifera* can increase DENV or CHIKV infection in *Ae. aegypti* due to the effect generated by the interaction between the P40 polypeptide, encoded by the bacterium, and prohibitin, a protein related to arboviral infection in mosquito cells [98, 99] (Fig. 2).

These results have opened a window for further research to understand mosquito–-microbiota interactions, their influence on arboviral infection in *Ae. aegypti* and *Ae. albopictus*, and the development of novel vector control strategies. Paratransgenesis stands out among the main strategies developed with the use of microbiota to control arboviral and vector-borne diseases [35]. This technique is based on the genetic manipulation of symbiotic bacteria to produce antipathogen effector molecules, followed by the reintroduction of the modified symbiont into the arthropod host to reduce vector competence [75]. Interestingly, genetic engineering research has evaluated molecules generated by “stable” species of the vector microbiota that are commonly present in different mosquito species (“core” microbiota).

Most of these studies have focused on persistent symbionts, capable of secreting antagonistic molecules that are horizontally and/or vertically transmitted, thus allowing self-sustainment of the modified symbionts in the field [36]. In addition, these symbionts must present the potential to survive long enough in the mosquito to ensure the effective and constant production of effectors that limit pathogen replication in the vector [35, 67, 84]. The primary investigations have studied genetic modifications of symbiont microbiota of insects such as *Rhodnius prolixus* to control the *Trypanosoma cruzi* parasite, the causative agent of Chagas disease, and *Anopheles* spp. for the control of the *Plasmodium* parasite, the causative agent of malaria [84]. In the case of *Aedes*, the bacterium *Asaia* is postulated as a promising option for arboviral control due to its ability to colonize both laboratory and field mosquitoes [35, 80, 82, 83, 100]. In addition, this bacterial species has been used in paratransgenesis for malaria control, demonstrating limitations in larval development in *Anopheles* spp. [35]. Despite the advances achieved, most studies are still in vitro trials due to the possible environmental risk that could be generated by the release of genetically modified organisms and the potential implications of interactions between modified microorganisms and native insect vectors.

Based on advances in next-generation sequencing, especially shotgun metagenomics, much better understanding of the complexity of the *Ae. aegypti* and *Ae. albopictus* microbiota in the presence or absence of viral pathogens is expected. Currently, it is important to move towards a deeper understanding of the inherent molecular mechanisms of interaction between the microbiota, the pathogens of *Aedes* mosquitoes, and their impact on the modulation of vectorial capacity. This new metagenomic next-generation sequencing (mNGS) approach can favor the detection of microorganisms that can be exploited to develop different applications for the efficient management of *Aedes* vectors and the diseases they transmit. In addition, considering that the population structure of *Ae. aegypti* and *Ae. albopictus* is strongly influenced by geography and the type of breeding site, the anthropogenic, environmental, and geographical factors that affect acquisition should also be considered in future studies and abundance of microbial communities. This information could be used to better understand the potential of the microbiome to prevent or increase mosquitoes' ability to transmit medically important arboviral pathogens, depending on the conditions of the habitat where these vector species circulate or coexist.

### Metagenomics and the rise of virome studies in mosquitoes

Metagenomic next-generation sequencing (mNGS), also known as shotgun deep-sequencing, is a high-throughput sequencing strategy with high efficiency and a short turnaround time [101]. mNGS is defined as “the application of the modern genomics without the need for isolation and laboratory culture of individual species” [102], and facilitates the understanding of the composition of all genetic material (DNA or RNA) in a clinical or environmental sample [103]. The deployment of mNGS has identified structural and functional diversity of vertebrates and invertebrates microbiomes [35, 78, 104]. In the case of viral communities in insects, even recently there was a “biased” conception of viruses as agents that cause disease; however, today, with the advances in metagenomics studies, the proper structural and functional diversity of insect viromes has begun to be elucidated [104–106]. Thus, metagenomics methods, especially during the last decade, have provided new insights into the complexity of insect-borne viruses [41, 107–109], relevant for active pathogen surveillance and response to emerging and re-emerging infectious diseases [37, 103, 110, 111].

Metagenomic studies in mosquito vectors are relatively recent. This technique is one of the most common sequencing approaches for the characterization of mosquito microbial communities and the description of phylogenetic relationships [35, 100, 112]. With this new approach, it has been possible to elucidate that *Ae. aegypti* and *Ae. albopictus* mosquitoes harbor a rich and diverse virome composed mainly of ISVs [39, 107, 113–115]. Unlike arboviruses, which have dual host tropism (vertebrates and arthropod vectors), ISVs replicate exclusively in insect populations and cannot replicate in vertebrate cells or infect humans [41, 116, 117]. Therefore, being naturally associated with arthropods, they can be considered members of viral communities of insects exclusively (viromes), and are not considered pathogens [104, 117]. In adult insects, the highest proportion of ISVs characterized so far correspond to RNA viruses [104] without causing apparent affectation, suggesting their high adaptation and relationship with insects [104].

**Fig. 3:**
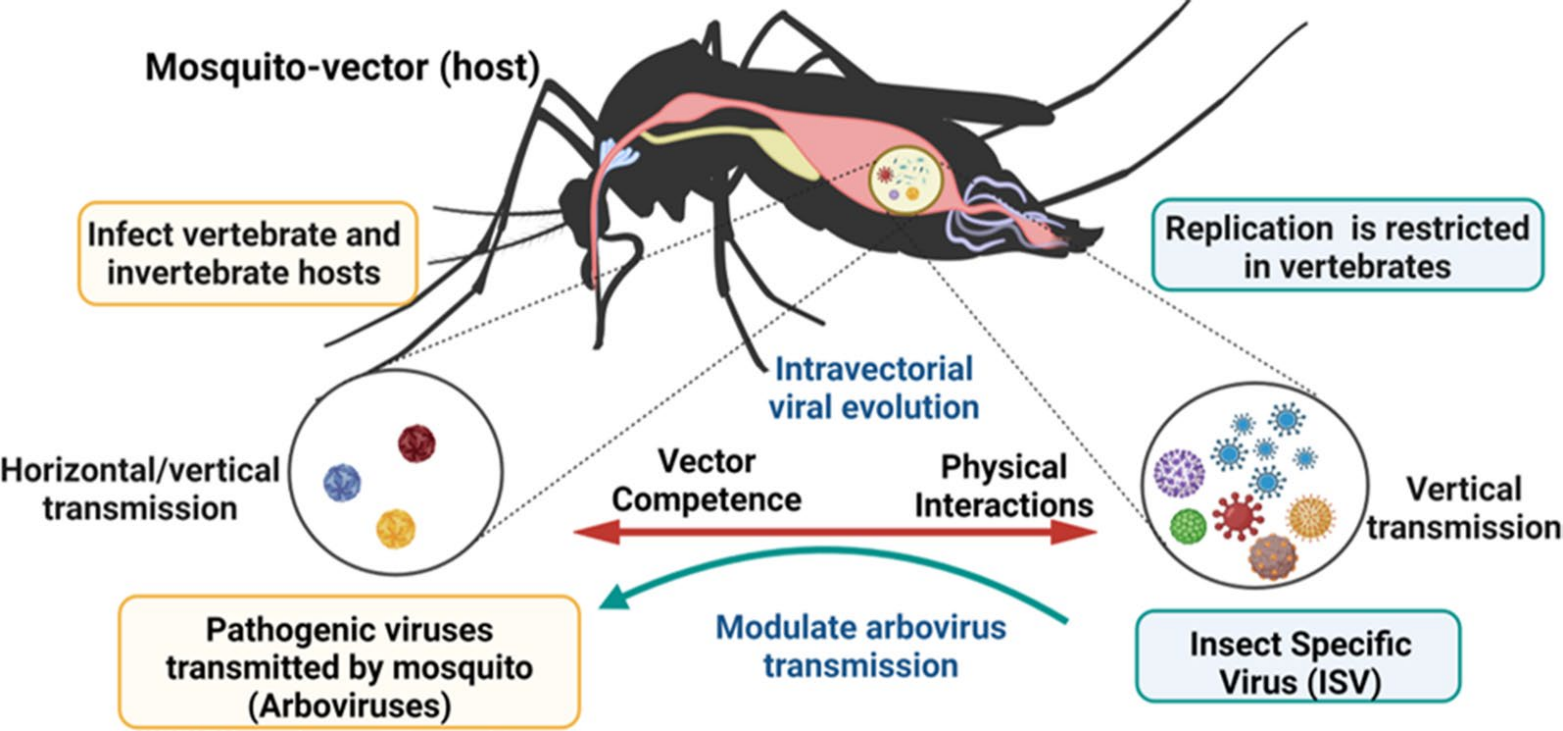
Schematic summary of some characteristics of the *Aedes* mosquito virome from metagenomic studies. The *Aedes* virome is formed by arboviruses and in greater proportion by insect-specific viruses (ISV). Arboviruses are pathogenic viruses that are transmitted to humans by mosquito vectors (dual host), while ISVs are viruses that replicate exclusively in insects and are not capable of infecting humans (single host). ISVs possibly evolved and diversified with their insect hosts. Some ISV species could potentially modulate vector competence in *Aedes* spp. Advances in vector viromics may contribute to the development of strategies to control and prevent arboviral diseases. Figure created with BioRender.com

Metaviromic analyses in mosquitoes carried out recently show a significant increase in the number of specific viral or ISV sequences, both in natural populations and in mosquito-derived cell lines [37, 116, 118]. These findings indicate a higher abundance and prevalence of ISVs in mosquito populations than in arboviruses in approximately 1–2% of individuals [34]. Research on ISVs in mosquito vectors has focused mainly on *Culex* spp. mosquitoes, *Aedes* spp., and *Anopheles* spp. [109, 115, 119–121]. Phylogenetic analyses on the reconstruction of ancestral traits in ISVs indicate that there are variations in the diversity and abundance of viruses between vector species; however, viral families such as *Flaviviridae* (positive-sense [+] single-stranded RNA [ssRNA]), *Bunyaviridae* (negative-sense ssRNA [−]), *Rhabdoviridae* (ssRNA), *Reoviridae* (double-stranded RNA [dsRNA]), and *Togaviridae* (+ssRNA) are shared [34, 37–39, 122]. These discoveries have opened a new view on the diversity and evolution of ISVs [38, 39, 116] and their influence on vectorial competence [123] for efficient transmission of human pathogens (arboviruses) [118], in addition to their potential use as biological control agents or new vaccine platforms [44, 45] (Fig. 3).

Phylogenetic analyses and experimental studies have shown that many ISVs isolated from mosquitoes contain ancient and diverse lineages, which possibly evolved and diversified with their host insects [122, 124]. Research on the reconstruction of ancestral traits in ISVs of *Bunyavirales* has evidenced a basal phylogenetic relationship between the dual-host bunyavirus and insect-specific ancestors [125]. The close relationship found between ISVs and human pathogenic arboviruses [38, 39, 122] has generated the hypothesis about the role they can play in modulating arboviral transmission [44, 45] (Fig. 3). ISVs are found at all stages of life in both male and female mosquitoes. This is associated with their efficient transmission to offspring (transovarial transmission) [104, 118] and coexistence with their insect host over a long period [39, 124]. In addition, there is evidence of the presence of viral RNA in the insect transcriptome and a high incidence of endogenous copies in the insect genome, suggesting a crucial role of ISV in the evolution of RNA viruses [126]. In this way, it could be inferred that, if ISVs are ancestral to arboviruses, they could be studied to understand the evolution from a single host to a dual host, as well as to elucidate the factors that influence the “*switch*” of viruses from arthropods to viruses with emerging potential.

### Virome in *Ae. aegypti* and *Ae. albopictus*

Metagenomics studies in *Ae. aegypti* and *Ae. albopictus* conducted in different geographical areas of the world (Americas, Asia, Africa, Europe, and Australia) [73, 107, 121, 127–134] have identified that *Aedes* host a viral community with high diversity. Currently, the available data focus mainly on *Ae. aegypti*, compared with the virome of *Ae. albopictus*, possibly because it is considered the primary vector of arboviruses and with a vast geographical distribution [23, 107, 114]. It should be noted that most of the viruses discovered lack a formal taxonomic classification (unclassified viruses), which limits the proper understanding of the diversity of the circulating virome in these vector species. This condition highlights the need to generate more robust databases allowing us to improve the viral characterization of mosquitoes that transmit infectious diseases.

**Fig. 4:**
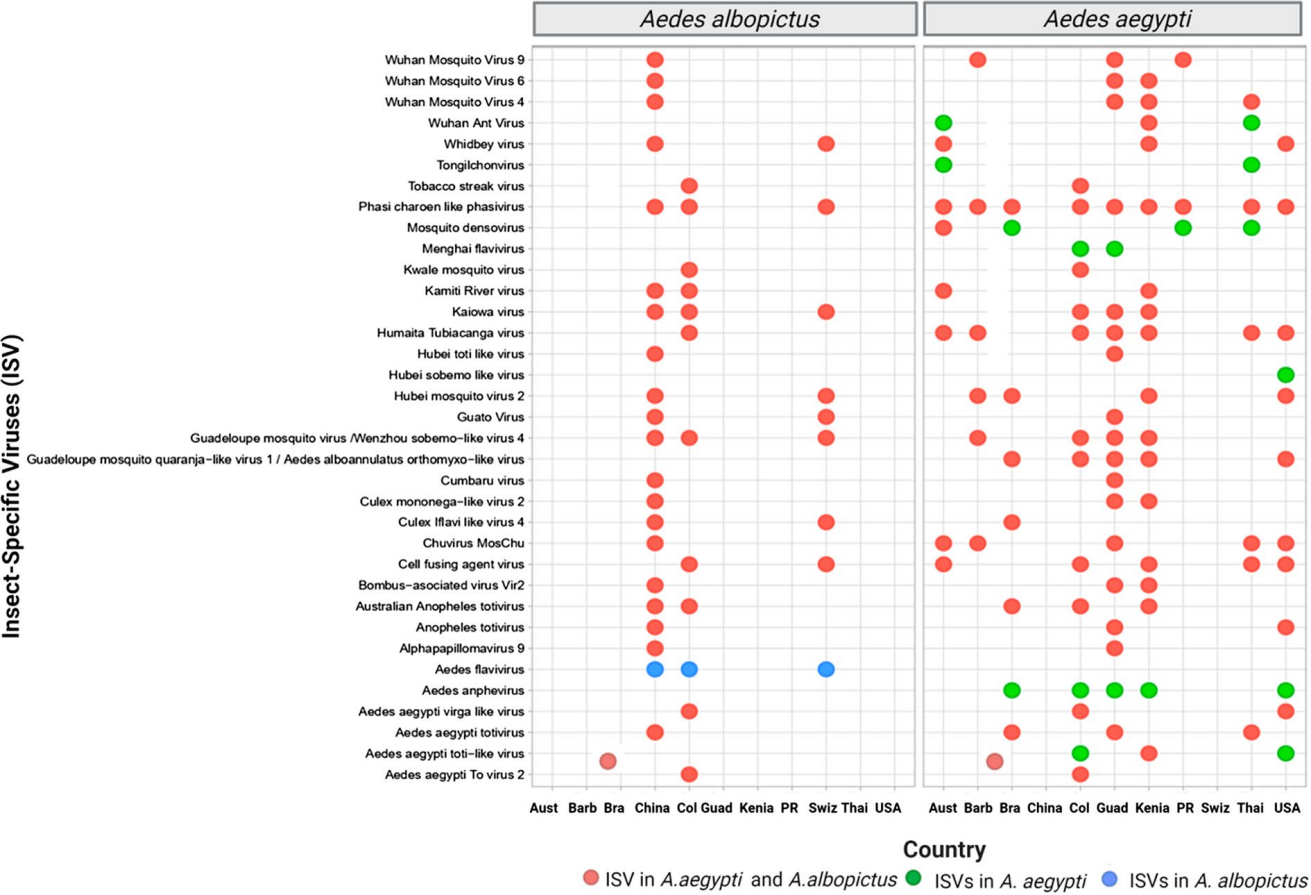
Conservation of insect-specific viruses (ISV) (virome) in *Ae. aegypti* and *Ae. albopictus* in countries of different geographical areas of the world. Pink circles indicate the presence of shared ISVs in *Ae. aegypti* and *Ae. albopictus*, yellow circles indicate unique ISVs in *Ae. aegypti*, and blue circles indicate unique ISVs in *Ae. albopictus*. *Aust* Australia, *Barb* Barbados, *Bra* Brazil, *Nig* Nigeria, *Guad* Guadeloupe, *Ken* Kenya, *PR* Puerto Rico, *Suiz* Switzerland, *Tha* Thailand, *USA* United States of America

Curiously, in these investigations, similarity in the composition of the virome has been evidenced in *Aedes* species, made up mainly of the families *Flaviviridae*, *Totiviridae*, *Phenuiviridae*, *Orthomyxoviridae*, *Virgaviridae*, and *Secoviridae*. In addition, this research highlights the conservation of viral species in the *Aedes* virome, such as Phasi Charoen-like virus (PCLV) (*Phenuiviridae*), Humaita-Tubiacanga (HTV) (unclassified viruses), Guadeloupe mosquito virus (GMV)/Wenzhou sobemo-like virus 4 (unclassified viruses), cell fusing agent virus (CFAV; *Flaviviridae*), Guadeloupe mosquito quaranja-like virus 1/Aedes alboannulatus orthomyxo-like virus (*Orthomyxoviridae*), and Australian Anopheles totivirus (*Totiviridae*) (Fig. 4), demonstrating the presence of a “core virome,” perhaps associated with its ecology, similar food sources, selective host pressures, and microbial interactions [67, 112]. Therefore, it is considered that the “core virome” in *Ae. aegypti* and *Ae. albopictus* likely plays a role in mosquito homeostasis and may also have implications in vector competition for arbovirus transmission [39, 118, 132]. Future studies are needed to fully understand these complex interactions.

In most metavirome studies carried out so far, the viral families *Flaviviridae*, *Orthomyxoviridae*, *Totiviridae*, and *Phenuiviridae* have been the most prevalent (Fig. 4). This suggests a possible origin from the ancestral *Aedes* mosquito and evolution with the vector in different parts of the planet. This is consistent with several authors that propose processes of co-evolution and diversification between ISVs and the insect [113, 116, 121, 135], as previously suggested for the families *Bunyaviridae*, *Flaviviridae*, and *Rhabdoviridae* [38, 123]. In particular, the family *Totiviridae* has been mainly associated with fungi and plants [136]. However, its prevalence has increased among arthropods, possibly due to horizontal virus transfer mechanisms [132]. According to Shi et al*.* [137], interspecies virus transmission is a joint event across the ISV landscape, and it has likely been a significant factor in the evolutionary history of such viruses. Considering this new vision on the virome of *Ae. aegypti* and *Ae. albopictus* mosquitoes, subsequent studies could focus their attention on investigating how these viruses could influence mosquito–virus–pathogen interactions in the dynamics of arbovirus–insect transmission.

Some investigations of viral communities in *Ae. aegypti* and *Ae. albopictus* also show a stable virome profile at different life stages (larva, pupa, and adult), grown in the laboratory and collected in the field [107, 127]. In this way, several studies suggest that the “core virome” can be acquired vertically (from parent to offspring) [127] or from the environment [127]. Accordingly, studies by Coatsworth et al*.* [132] confirmed the vertical transfer of ISVs in *Ae. aegypti* by metagenomic studies conducted over several generations. Similarly, Thannesberger et al. [128] showed that the local ecosystem could play a preponderant role in the composition of viral communities in mosquito vectors. Interestingly, most metaviromic analyses in *Aedes* mosquitoes collected from the field show the presence of a “core virome” with more extraordinary diversity than the virome of laboratory mosquitoes. This is probably associated with the variable geographical and environmental conditions found in nature versus the standardized conditions of water and resources available in the laboratory [37, 129, 130]. The evident contrast between the scenarios in which field and laboratory mosquitoes are exposed could reflect the viral diversity of their respective environments [132]. Thus, future investigations could identify the impact of different biological and environmental variables on ISVs.

Comparative analyses between the virome of *Ae. aegypti* and *Ae. albopictus* show differences in viral composition and diversity. Metagenomic studies show a higher ISV richness in *Ae. aegypti* [73, 107, 128, 131, 132], related to its high susceptibility to arbovirus infection [23, 29, 63]. In contrast, the virome of *Ae. albopictus* presents a wide diversity of viruses associated with vertebrates, insects, plants, bacteria, and fungi [24, 127, 129, 130], which can probably be explained by the different cycle, ecotope, and environmental factors, including breeding and feeding sites [29, 80]. In addition, it must be highlighted that specific viral species have been identified for each vector and geographical region. However, unique ISVs have been detected in Aedes anphevirus (AeAV; *Xinmoviridae*) and Aedes aegypti totivirus (*Totiviridae*), while in *Ae. albopictus* only the ISV Aedes flavivirus (AeFV; *Flaviviridae*) has been found. These differences in the virome composition of these vectors could reflect essential differences in evolutionary history and host immune responses [78, 81] and differences in virus–mosquito interactions, potentially related to vector competence [35, 45, 112].

### ISVs seems to modulate arbovirus infections in *Ae. aegypti and Ae. albopictus*

Considering the role of some symbiont members of the microbiota of insect vectors, it is considered that some ISVs may also have a similar effect on vector competition by suppressing or enhancing arbovirus replication in insect vectors [45, 104, 111, 117]. Some authors have proposed that mosquito-associated ISVs could have potential applications as (i) biological control agents against vector-borne diseases, (ii) diagnostic therapies, and (iii) new vaccine platforms [34, 35, 44, 45, 104, 117, 118].

The first characterized ISVs belong to the genus *Flavivirus*, as the CFAV, isolated from a culture in a cell line of *Ae. aegypti* [138]. This virus can also replicate in *Ae. albopictus* cell lines; however, it does not show a cytopathic effect on invertebrate cell lines. Recent studies by Zhang et al*.* [139] found that CFAV infection significantly improved DENV replication, possibly due to an increase in the expression of ribonuclease kappa (RNASEK), known to promote infection of endocytosis-dependent viruses and pH-dependent entry. The authors indicate that increased CFAV-induced RNASEK expression will likely contribute to improved DENV replication in CFAV-infected cells [139].

Research conducted by Schultz et al*.* [42] examined cell lines of *Aedes* species. The suppression of the arbovirus in the presence of ISV CFAV and PCLV demonstrates that dual ISV infection managed to decrease the growth of ZIKV, DENV, and La Crosse encephalitis virus (LACV) by up to 90% in immunocompetent cells of *Ae. albopictus* and *Ae. aegypti*. Another study characterized Aedes anphevirus (AeAV), a negative-sense RNA virus of the order *Mononegavirales*, capable of infecting laboratory colonies, wild mosquitoes, and cell lines of *Ae. aegypti* and *Ae. albopictus* worldwide [140]. It was identified that *Ae. aegypti* cells, co-infected with AeAV and *Wolbachia*, improved AeAV replication and slightly reduced DENV replication in vitro [140]. These data suggest that there are mechanisms of viral competition [141] as exclusion by superinfection. They may involve competition or modification of cellular resources that reduce receptor binding, viral entry, RNA replication, and translation of the secondary virus [42, 45, 142] or mechanisms of both positive and negative regulation of the antiviral immune response of the vector [39].

A similar study evaluated Nhumirim virus (NHUV) (*Flaviviridae*) pre-inoculated or inoculated simultaneously with ZIKV and dengue-2 virus (DENV-2) in *Ae. albopictus* cells, showing a significant reduction of these viruses [43]. Additionally, trials with individuals of *Ae. aegypti* also demonstrated decreased ZIKV infection rates in mosquitoes inoculated with NHUV compared with those not exposed [43]. Likewise, in *Culex quinquefasciatus,* a decrease in the transmission rates of West Nile virus (WNV) was observed when the vector was previously exposed to NHUV [143]. These results indicate that some ISV species could modulate vector competition in *Aedes* spp. and *Culex*.

On the other hand, Nazar et al*.* [144] investigated the ability of Eilat virus (EILV), an ISV of the *Togaviridae* family, to interfere with viruses of the same family, such as Sindbis virus (SINV), CHIKV, western equine encephalitis viruses (WEEV), eastern equine encephalitis virus (EEEV), and Venezuelan equine encephalitis virus (VEEV). In vitro results in *Ae. aegypti* C7/10 cells showed that EILV infection reduced replication of pathogenic viruses regardless of virus or multiplicity of infection. In addition, in vivo trials in *Aedes* mosquitoes pre-inoculated with EILV and fed blood containing CHIKV also showed a reduction in the rate of infection and the spread of CHIKV [144]. These results suggest different interactions between ISVs and arboviruses, such as competitive inhibition and superinfection exclusion [123]. However, it should be noted that studies on the relationship between ISV–arbovirus–microbiota of insects are only just emerging. Therefore, new investigations are needed to better understand this type of interaction, which may result in the development of new approaches for the control and prevention of arboviral transmission.

## Conclusions

*Aedes aegypti* and *Ae. albopictus* mosquitoes that transmit viruses of medical and economic importance, such as DENV, CHIKV, and ZIKV, among others, continue to be a major threat to global public health. Currently, the geographical spread of these mosquito vectors, and thus of arboviruses, is increasingly extending to new regions under the influence of multiple social, environmental, and ecological factors. In recent years, shotgun metagenomic sequencing has improved our understanding of the composition and, in part, the functionality of the *Aedes* microbiota/virome. These advances have revealed the critical role that some of the resident microorganisms play in host fitness, especially in the presence and absence of viral pathogens. It is known that *Ae. aegypti* and *Ae. albopictus* share a core set of microorganisms, especially bacterial and viral. However, many aspects of this area of knowledge remain unclear. Therefore, understanding the true diversity of the mosquito microbiome and its interactions with the host, as well as its dynamics in the face of arbovirus infection events, is critical, given the evidence as potential biological control agents. Recent metavirological analyses indicate that the “core virome” of *Ae. aegypti* and *Ae. albopictus*, composed mainly of ISVs, has the potential to alter the susceptibility of mosquitoes to certain arboviruses, as well as to show evolutionary relationships with these pathogens. Further studies, especially of ISVs, including isolation, whole-genome sequencing, and even functional assays, are thus warranted to better understand their origins, pathogenic potential, molecular mechanisms affecting vector competence, and potential biotechnological applications.

It is therefore emphasized that microbiota–ISV–arbovirus interactions in the mosquito (host) form an ecologically complex system, influenced by the geographical and environmental conditions to which the vector is exposed. For this reason, future analyses should also consider the anthropogenic, environmental, and geographical factors that influence the acquisition and abundance of the microbial communities of *Ae. aegypti* and *Ae. albopictus*, and identify the change in the composition and diversity of the microbiota/virome in the different life stages of *Aedes* (pupa, larva and adult), at both the field and laboratory levels. This information could be used to better understand the potential of the microbiota to prevent or enhance the mosquito's ability to transmit arboviral pathogens of medical importance, depending on the habitat conditions where these vector species circulate or coexist. Finally, it is concluded that the path is open for further strengthening of omics and bioinformatics sciences, towards a better understanding of *Aedes* insect biology and arbovirus epidemiology, and the development of potential “novel” arboviral intervention strategies.

## Data Availability

Not applicable.

## Abbreviations

ISV: Insect-specific viruses
AMP: Antimicrobial peptides
CHIKV: Chikungunya virus
DENV: Dengue virus
DDT: Dichlorodiphenyltrichloroethane
miRNA: MicroRNA
MEC: Midgut epithelial cells
PCR: Polymerase chain reaction
P40: Polyvinentide P40
Pr: Prohibitin.
ROS: Reactive oxygen species
ZIKV: Zika virus
